# Synthesis of Fluorescent, Small, Stable and Non-Toxic Epitope-Imprinted Polymer Nanoparticles in Water

**DOI:** 10.3390/polym15051112

**Published:** 2023-02-23

**Authors:** Perla Benghouzi, Lila Louadj, Aurélia Pagani, Maylis Garnier, Jérôme Fresnais, Carlo Gonzato, Michèle Sabbah, Nébéwia Griffete

**Affiliations:** 1 Physico-Chimie des Electrolytes et Nanosystèmes InterfaciauX (PHENIX), Sorbonne Université, CNRS, 4 Place Jussieu, 75005 Paris, France; 2 Saint-Antoine Research Center (CRSA) INSERM, CNRS, Sorbonne Université, 75012 Paris, France; 3 Laboratory for Enzyme and Cell Engineering UMR 7025, CNRS, Université de Technologie de Compiègne, Rue du Docteur Schweitzer, 60203 Compiègne, France

**Keywords:** molecularly imprinted polymer, nano isothermal titration calorimetry, epitope, adsorption, toxicity

## Abstract

Molecularly imprinted polymers (MIPs) are really interesting for nanomedicine. To be suitable for such application, they need to be small, stable in aqueous media and sometimes fluorescent for bioimaging. We report herein, the facile synthesis of fluorescent, small (below 200 nm), water-soluble and water-stable MIP capable of specific and selective recognition of their target epitope (small part of a protein). To synthesize these materials, we used dithiocarbamate-based photoiniferter polymerization in water. The use of a rhodamine-based monomer makes the resulting polymers fluorescent. Isothermal titration calorimetry (ITC) is used to determine the affinity as well as the selectivity of the MIP for its imprinted epitope, according to the significant differences observed when comparing the binding enthalpy of the original epitope with that of other peptides. The toxicity of the nanoparticles is also tested in two breast cancer cell lines to show the possible use of these particle for future in vivo applications. The materials demonstrated a high specificity and selectivity for the imprinted epitope, with a K_d_ value comparable with the affinity values of antibodies. The synthesized MIP are not toxic, which makes them suitable for nanomedicine.

## 1. Introduction

“Precision therapeutic targeting” makes all medicine better: indeed, delivering the right dose of drugs to the right location in the body opens many doors. Hence, an intense prominence needs to be placed on the development of strategies that selectively and preferentially deliver therapeutic agents to the target site, simultaneously reducing the access to non-target sites. Such “targeting” is performed through the functionalization of nanoparticles with antibodies [1] or aptamers [2] in order to increase the efficiency of immunotherapy and the therapeutic response [3].

Only recently, molecularly imprinted polymers (MIPs) have been used to confer “targeting properties” to nanoparticles [4]. While MIPs are well-established materials in fields such as sensors [5,6], their in vivo use for cancer therapy is only at its early stages, and their use in clinical trials is essentially nonexistent [7,8,9]. MIPs are synthetic materials that exhibit excellent binding properties with affinity and selectivity comparable to those of antibodies [10,11]. Their imprinting process consists in polymerizing suitable amounts of a functional monomer and a cross-linker in the presence of a molecule of interest (i.e., the target). After the extraction of such a molecule, the polymer matrix contains binding sites which are perfectly complementary to the target in terms of size, shape and position of the interacting groups. MIPs, which can be synthesized easily and at a lower cost compared to antibodies, present good biocompatibility, solubility, the ability to cross the cell membrane and a lack of immunogenic response [12].

Due to all these features, MIPs are then highly regarded as suitable candidates for targeted cancer therapy. For example, MIPs have been successfully employed by Cecchini et al. [13] to target specific cells in zebra fish embryos, as well as by Koide et al. to in vivo inhibit the action of the human vascular endothelial growth factor [14]. Recently, Piletsky et al. developed imprinted nanoparticles against epidermal growth factor receptors capable of specifically recognizing the overexpressed protein at the surface of cancer cells [1].

However, even though MIPs have now shown their potential and suitability for targeted therapy, there is still room for the development of easy synthetic pathways. Indeed, a great majority of the current examples lie on the use of heterogeneous systems based on epitope-functionalized beads immersed in a pre-polymerization medium [15,16,17,18]. Upon synthesis, high-affinity MIPs are then eluted from the column and recovered. Even though automated systems have been developed [19], easy ways to synthesize small, stable and possibly fluorescent MIPs for targeting proteins using epitopes are still limited. The requirements to obtain such particles are indeed quite strict, including the need of aqueous media as solvents and only moderate heating during the polymerization step, in order to avoid epitope denaturation; in addition, the resulting particles must have an overall size lower than 200 nm for in vivo applications [20], as well as fluorescent properties for fluorescent imaging.

**Scheme 1:**
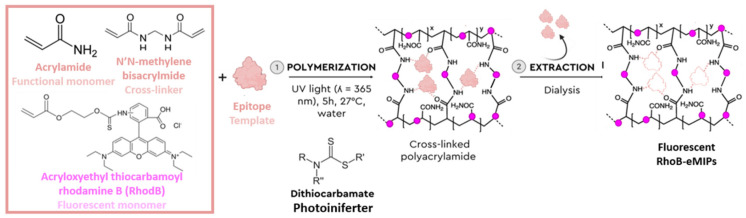
The MIP was prepared by co-polymerization (1) of monomers and a cross-linker in the presence of the target epitope, which acts as a molecular template. Upon extraction (2), the so-formed material retains molecular cavities, complementary in size, shape and functionality to the template and ready to re-bind it through weak interactions.

We report herein, the synthesis of a small, fluorescent, water-soluble and water-stable MIPs capable of specific and selective recognition of its target epitope via the use of a water-soluble, dithiocarbamate-based photoiniferter (Scheme 1). To the best of our knowledge, this is the first time that a water-soluble photoiniferter is applied to the synthesis of a fluorescent, epitope-imprinted MIP in an aqueous medium. This technique was applied to set the MIP binding parameters in terms of affinity and selectivity.

The binding properties of the MIP were studied by nanoITC [21,22] with respect to the epitope X, used in the synthesis, as well as to Y and Z, which have a different sequence compared to X (Table 1). Non-imprinted polymer nanoparticles (NIPs) without any imprint were also used to show the MIP specificity toward its imprinted epitope. Finally, the toxicity of the MIP was tested in MCF7 and SUM-159 breast cancer cell lines.

## 2. Materials and Methods

### 2.1. Chemicals

Sodium diethyldithiocarbamate trihydrate (NaDTC), sodium chloroacetate, 6% hydrochloric acid, acrylamide (AAm), N,N-methylene-bis-acrylamide (MBAm) and phosphate-buffered saline (PBS) were from Sigma (Saint-Quentin Fallavier, France). Acryloxyethyl thiocarbamoyl rhodamine B (fluorescent monomer) was obtained from Polysciences. Methanol was from VWR Chemicals (Strasbourg, France). Acetic acid was from Carlo Erba. Deionized water (resistivity ≥ 18.2 MΩ cm^−1^) was obtained using a Milli-Q plus unit (Millipore, Molsheim, France). CDCl_3_ was from Eurisotop (Saint-Aubin, France).

### 2.2. Synthesis of the Photoiniferter 2-(N,N-diethyldithiocarbamyl) Acetic Acid (DCAA)

The synthesis of DCAA was adapted according to Sunayama et al. [23]. Briefly, in a 50 mL glass flask sealed with a cap, NaDTC (8 mmol) was dissolved in water (10 mL) under magnetic stirring. After complete dissolution, sodium chloroacetate (8 mmol) in water (10 mL) was added dropwise, and the resulting mixture was left under stirring at room temperature for 2 days, protected from light with aluminum foil. The reaction flask was then cooled on ice, and the pH of the solution was adjusted to 1.5 with conc. HCl. DCAA appeared as a white precipitate which was collected by filtration, washed with a little water and dried overnight at 50 °C to obtain a white solid with 90% yield. 1 H NMR (CDCl_3_): 1.28 to 1.36 ppm (m, 6H), 3.76 to 4.06 (2 q, 4H), 4.21 (s, 2H).

### 2.3. Synthesis of MIP and NIP

We dissolved 1.40 mmol of acrylamide, 3.24 mmol of the cross-linking agent, N,N-methylene-bis-acrylamide and 2 µmol of fluorescent monomers rhodamine B in 1.5 mL of PBS (pH = 7, 1X). Then, 0.150 mg of epitope X was added. The pre-polymerization complex was then allowed to self-assemble under orbital stirring at 250 rpm for 1.5 h. Then, 8.3 × 10^−4^ mmol (0.83 µmol) of DCAA was added to the reaction medium. The mixture was purged with nitrogen for 10 min, and the polymerization was allowed to proceed under UV irradiation at 365 nm (UVA lamp, UVC 13728- Pierron, 7 W/cm^2^) for 5 h. The final product was thoroughly washed with deionized water using a dialysis membrane (12–14 kD) to remove unreacted monomers and unbound epitope. The MIP was further dialyzed with a mixture of methanol/acetic acid/water 4.5/1/4.5 (in volume) and distilled water to remove the bound epitope. The non-imprinted polymer (NIP) was synthesized using the same protocol but without the epitope in the mixture.

### 2.4. nanoITC, FTIR, DLS, TEM, Fluorescence

The size and size distribution (diameter D, polydispersity σ) of MIP and NIP are characterized using a JEOL-100 CX transmission electron microscope. A droplet of the aqueous diluted nanomaterial dispersion was deposited on a carbon-coated copper grid and dried at room temperature for at least 5 h before transmission electron microscopy (TEM) observations. Infrared spectra were obtained on a Bruker Tensor 27 spectrometer on pressed KBr pellets. The spectra were obtained at regular time intervals in the MIR Region of 4000–400 cm^−1^ at a resolution of 4 cm^−1^ and analyzed using OPUS software. The hydrodynamic diameters (Dh) were recorded using a Malvern Instruments Nanosizer. NanoIsothermal titration calorimetry was run on a NanoITC TA instrument. The spectra were analyzed using NanoAnalysis software. Fluorescence images were obtained with a ZEISS microscope and analyzed with the software ImageJ.

## 3. Results and Discussion

**Figure 1:**
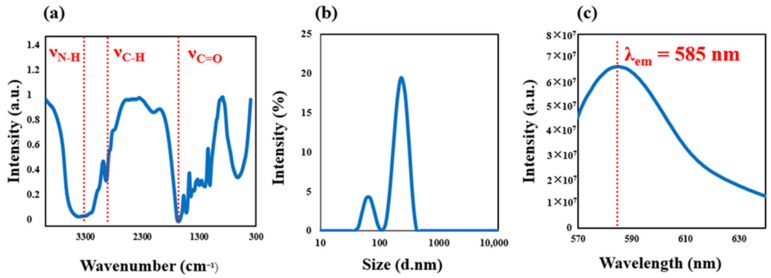
(**a**) Fourier transform infrared spectroscopy (FT-IR) spectra; (**b**) dynamic light scattering (DLS); (**c**) fluorescence spectra.

The study of the size, morphology and surface functionalization of MIP nanoparticles is essential for assessing the efficiency of their synthesis. For this, we used different techniques such as dynamic light scattering (DLS), TEM and infra-red spectroscopy. By Fourier transform infrared (FT-IR) spectroscopy of the MIP (Figure 1a) we detect vibration bands at 1660 cm^−1^ (C=O stretching), 2900 cm^−1^ (C–H stretching) and 3350 cm^−1^ (N-H stretching), which are all consistent with a p(AAm-co-MBAm)-based backbone. These peaks then accounted for the successful polymerization of AAm and MBAm upon UV irradiation. Dynamic light scattering measurements indicate that such MIP particles have an average hydrodynamic diameter of around 200 nm with a polydispersity index of 0.4993 (Figure 1b); however, the presence of two peaks indicates a polydisperse sample. In particular, a minor peak below 100 nm suggests that the amount of such small particles is quite high (it is indeed well known that the intensity-weighted, size distribution by DLS is dominated by large particles). DLS measurements were also performed 2 months after the synthesis (Figure S1), thus confirming the stability of MIP over time.

**Figure 2:**
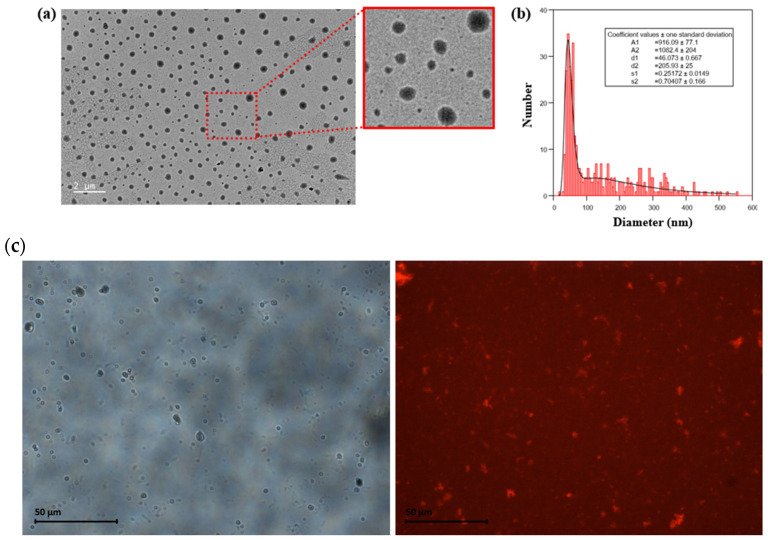
(**a**) Transmission electron microscopy images of MIP at 20,000× magnification. The spherical MIP particles have a size distribution ranging from 20 nm to 300 nm; (**b**) size distribution of MIP as acquired from TEM; (**c**) representative fluorescence microscopy images with (right) and without (left) the fluorescent filter.

The particles were imaged by TEM, and the obtained pictures showed that a great majority of particles has a size below 100 nm (Figure 2a,b). The sizes measured by TEM were in line with those obtained by DLS. Eventually, fluorescence analysis showed that upon excitation at 540 nm, an aqueous dispersion of MIP emitted an intense fluorescence at 585 nm, typical of the rhodamine dye (Figure 1c). The observation of these particles under a fluorescence microscope confirms that the rhodamine monomer had been successfully incorporated into the particles (Figure 2c).

**Scheme 2:**
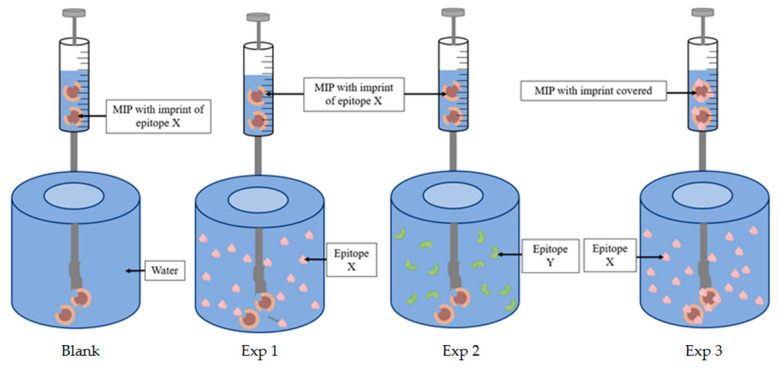
Schematic representation of the different nanoITC experiments with the MIP. The imprinted polymer is loaded into the syringe placed in a very precise injection device. The injection device was inserted into the sample cell containing a solution of epitopes.

In order to estimate the MIP affinity toward the epitope, nano isothermal titration calorimetry (nanoITC) is used. The ITC was performed at 25 °C with a thermal activity-controlled calorimeter equipped with a high-performance titration unit, as shown in Scheme 2. Before each measurement, each solution and suspension were degassed to remove the dissolved air bubbles. In total, 950 µL of epitope solution was titrated by adding 25 × 10 µL aliquots of a MIP suspension at a concentration of 1.9 mg/mL. The first injection, set at 5 µL, was not taken into account in the titration, due to the presence of a drop at the edge of the syringe which could distort the result. To compensate the effect of dilution of the MIP, the titration was also systematically conducted into the solvent of the epitope solution. The data were analyzed using the NanoAnalyze software provided by TA instruments. The heat measured during a calorimetric titration is proportional to the molar ratio of the bound ligands. Scheme 2 shows the different steps of the experiments we carried out. Experiment 1 represents the titration of the MIP with the epitope X, and experiment 2 represents the MIP with the epitope Y, that was not used during the synthesis of the MIP and was sufficiently different from epitope X to have a lower affinity for the MIP.

**Scheme 3:**
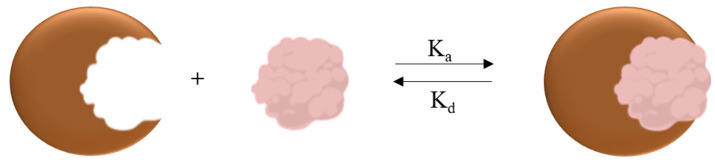
Molecularly imprinted polymer and epitope reaction scheme with *n* = 1 stoichiometry.

The hypothesis we considered is that the adsorption of an epitope molecule onto its imprint should be equimolar (one epitope per imprint) and that this principle would support at best the adsorption process (Scheme 3). This model does not consider the non-specific binding of epitopes. Hence, we synthesized a non-imprinted polymer and tested the adsorption of the epitope.

**Figure 3:**
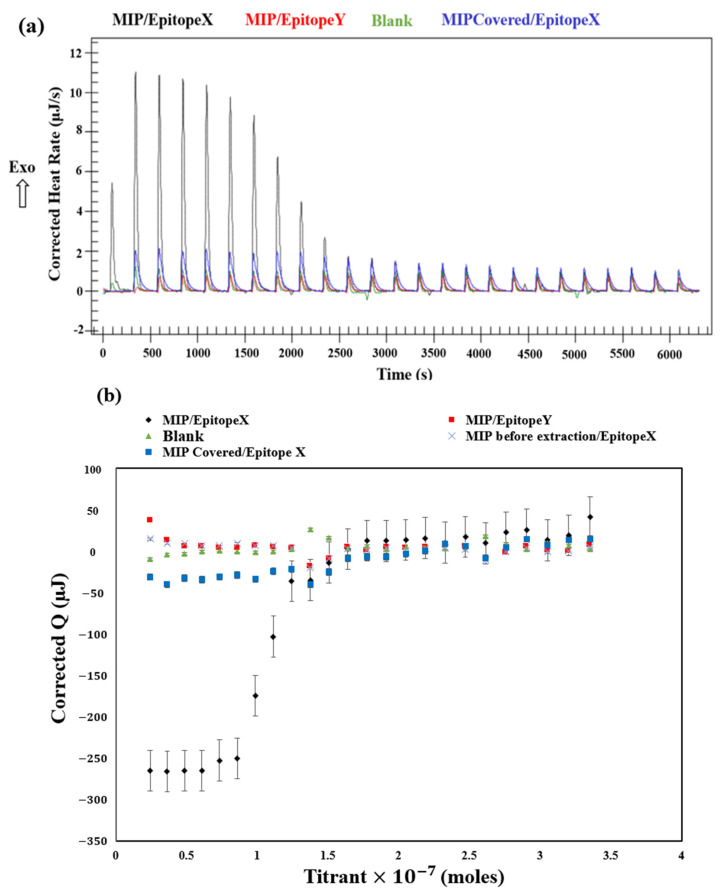
Calorimetric data for the binding kinetics between MIP and epitope X (black), MIP and epitope Y (red), covered MIP and epitope X (blue), MIP and water (green) and MIP before epitope extraction and epitope X. (**a**) Raw data from exothermic binding after continuous titration of MIP in the epitope solution. (**b**) Results calculated from the raw data as a function of the molar ratio of MIP to epitopes (the first value was excluded from the analysis).

Figure 3 shows the heat measured upon titration of the MIP suspension into a solution of epitope at 40 µM. Each injection step increases the number of MIP particles into the measurement cell, thus the occurrence of binding events. The blank allows the distinction of the heat resulting from epitope–cavity binding from the heat associated with MIP dispersion dilution. The blank titration (green) demonstrates a constant endothermic energy, which is classical in ITC titration when dilution only occurs. The black curve in the Figure shows higher peaks, confirming the adsorption of the epitope onto the MIP following a simple adsorption on independent sites. However, to determine if the targeting is specific for the epitope, an identical experiment was performed by modifying the X epitope solution with another epitope, Y (red), that was significantly different in amino-acid sequence to ensure a different tridimensional structure, not recognizable by the spatial organization of the bridging chemical functions. The titration demonstrates that the heat is equal to the one obtained for the blank. This was due either to no interactions between the MIP and the Y epitope, or to a non-detectable energy of non-specific adsorption of epitope Y onto the MIP surface due to unorganized anchoring groups on the MIP surface. This result confirms the specificity of the MIP for the imprinted X epitope that was used for the synthesis of the imprints and the non-detectable enthalpy of non-specific interactions that might be compensated by entropic processes such as water or counter ions release. The amount of heat measured during the addition of the ligand can be represented by the following equation:

$$Q=\frac{V_{0}\times \Delta H_{b}\times \left[E\right]\times K_{a}\times \left[M\right]}{\left(1+K_{a}\times \left[M\right]\right)\times \left[X\right]}$$

where V_0_ is the cell volume, ΔH_b_ is the enthalpy of binding per mol of ligand, [E] is the total epitope concentration, K_a_ is the association constant, and [M] is the concentration of the MIP (or the free binding site concentration).

Integration and normalization of the peaks yielded the observed heat, Q_obs_, as a function of the molar ratio of the template and the MIP binding sites.

The integration of the peaks confirmed that nothing could be detected when epitope Y replaced epitope X in contrast to the results obtained with the dilution test of MIP into the epitope solvent. Another epitope sequence, epitope Z (Table 1), was used to confirm the recognition of X. The same conclusion as those for epitope Y is achieved, as no significant enthalpy higher than that of the dilution process could be detected (Figure S2).

The values obtained for the binding affinity (K_d_) and the enthalpy of binding (ΔH) were 6.003 × 10^−7^ ± 5.8 × 10^−7^ (M) and −26.63 kJ/mol, respectively (Table 2). The enthalpy value of the MIP towards its epitope X is negative, i.e., in favor of the anchoring of the epitope X into its specific imprints. The hypothesis of an equimolarity is confirmed here (*n* = 1). The integration of the peaks confirms that nothing happens in the presence of only water or in the presence of the Y and Z epitopes, in sharp contrast with the results obtained with the X epitope for which we measured a heat rate until the epitope was saturated. This confirmed the selectivity of the MIP towards the epitope used to make the imprints.

To further prove the specific adsorption of the epitope X onto the MIP, we saturated the MIP with the epitope and then measured the heat released after further addition of epitope using nanoITC (Experiment 3, Scheme 2). As it can be seen in Figure 3 (blue), the heat is practically similar to those one obtained for the blank and with the Y epitope.

As detailed in the experimental section, two dialysis steps were performed: the first to extract unreacted residues, and the second to extract the epitope bound to the MIP in order to free the imprints. Thus, to prove that specific adsorption of the epitope only occurs onto its imprint, we tested the particles before and after extraction of the epitope. As can be seen in Figure 3 (bottom figure), the heat measured for the sample before the second dialysis step (i.e., before the epitope removal) is low, similar to that measured for the blank. The small difference that we observed can be due to the fact that we did not saturate the MIP with the epitope and that there remained a small amount of imprint. On the other hand, the sample after the second dialysis step (i.e., with the imprints available) releases a high heat, typical of the binding of the epitope to the imprint. This experiment confirms the presence of the epitope-specific imprint.

**Figure 4:**
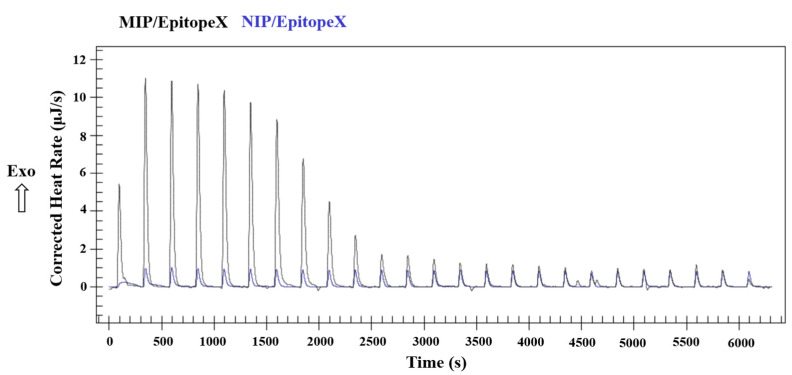
Calorimetric data for binding kinetics between MIP/epitope X and NIP/epitope X.

The nanoITC experiment using NIP nanoparticles with epitope X shows a similar result to that obtained in the blank experiment, evidencing no reactions (Figure 4) and the specificity of the synthesized nanoparticles.

**Figure 5:**
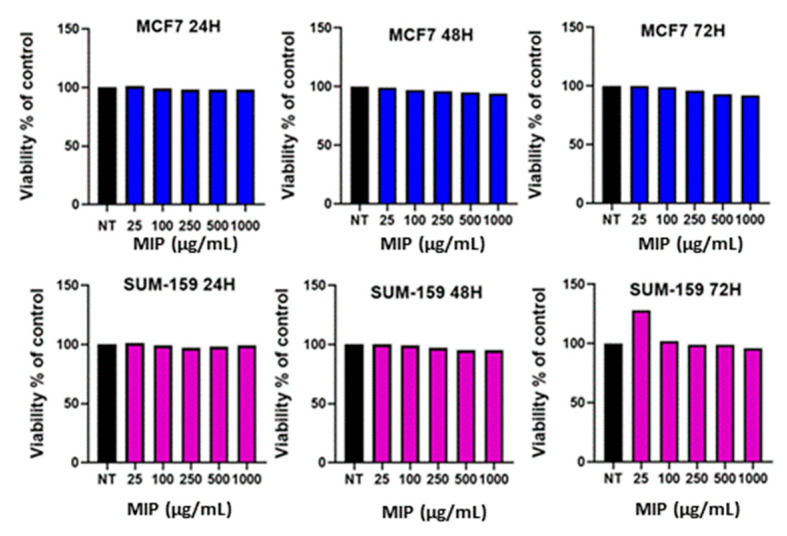
Cell viability in the two breast cancer cell lines MCF7 and SUM-159 after 24 h, 48 h and 72 h in the presence of increasing concentrations of MIP (NT = non treated).

The potential toxicity of MIP toward two breast cancer cell lines, MCF7 and SUM159, was subsequently assessed by the Celltiter-Glo^®^ viability assay (Promega, Madison, WI, USA). Briefly, the cells were incubated with increasing concentrations of MIP, and their viability was evaluated after 24 h, 48 h and 72 h of exposure. The results obtained are shown in Figure 5. As we can see, there is no decrease in cellular viability after MIP exposure compared to the untreated control (black column) in both models. Indeed, even at high concentrations, cellular viability remained >90%, proving the non-toxicity of the synthesized MIP nanoparticles. These experiments demonstrate the biocompatibility of MIP with living cells.

## 4. Conclusions

To conclude, epitope-specific molecularly imprinted polymers are promising synthetic materials that exhibit excellent binding properties toward the imprinted epitope, with affinity and selectivity comparable to those of antibodies. We synthesized herein fluorescent, small (below 200 nm) and well-stable epitope-imprinted polymer nanoparticles by photoiniferter polymerization in water. The materials synthesized with this method demonstrate a high specificity and selectivity for the imprinted epitope, with a K_d_ of 6 × 10^−7^ M, which is comparable to the affinity values of antibodies. The MIP is specific and selective to the imprinted epitope, as the NIP shows no binding affinity for the imprinted epitope, and the MIP shows no affinity for other epitopes than the one imprinted. The synthesized MIP proved to be nontoxic, as it does not reduce cell viability even at high concentrations, which makes them suitable for nanomedicine. As this synthetic approach has proved to be robust toward the imprinted epitope, we strongly believe that it could also apply to a whole new range of other proteins or epitopes, which could be helpful for biomedical as well as analytical applications. As more and more works dealing with epitope-specific, molecularly imprinted polymers for nanomedicine applications are published, having a large and long-lasting impact on the materials, nanomedicine and the polymer communities, we think that the approach here described could be largely used and adapted to other epitopes than the one used here.

## Figures and Tables

**Table 1:** Sequences of the epitopes used for the imprinting (X) and for the selectivity study (Y) and (Z).

Sequence	Peptide Structure
YRVRFLAKENVTQDAEDNC Labelled X	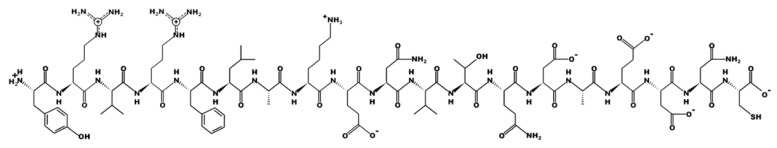
CNLAVAAASHIYQNQFVQ Labelled Y	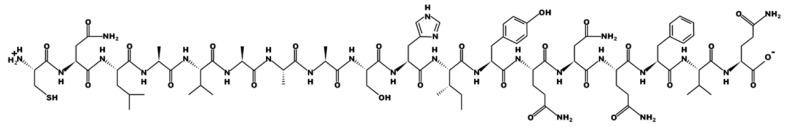
CQMILNSLINKSKSSMFQV Labelled Z	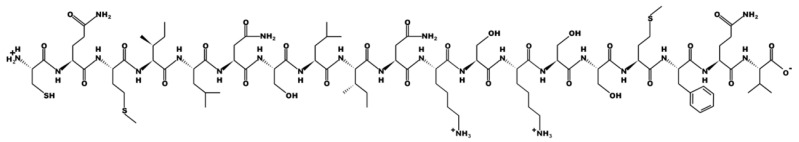

**Table 2:** Enthalpies and dissociation constant values obtained by fitting the data in Figure 3.

Template	ITC Probe	ΔH (kJ/mol)	ΔG (kJ/mol)	K^D^	*n*
X	X	−26.63 ± 0.036	−35.51	6.003 × 10^−7^ ± 5.8 × $10^{-7}$	1 ± 0.043

## Data Availability

The data presented in this study are available on request from the corresponding author.

## Supplementary Materials

The following supporting information can be downloaded at: https://www.mdpi.com/article/10.3390/polym15051112/s1, Figure S1: Dynamic light scattering (DLS) of the MIP after 2 months; Figure S2: Calorimetric data for the binding kinetics between MIP and epitope X and between MIP and epitope Z from the same protein.
