# Supplementary material for: Synthesis of Fluorescent, Small, Stable and Non-Toxic Epitope-Imprinted Polymer Nanoparticles in Water
Source: Polymers (Basel). 2023 Feb 23;15(5):1112. doi: 10.3390/polym15051112 (PMC10007256; doi:10.3390/polym15051112)
Supplement: Supplementary file 1 [file polymers-15-01112-s001.zip › polymers-2237640-supplementary.pdf]

Supporting information

Synthesis of fluorescent, small, stable and non-toxic epitope imprinted polymer nanoparticles in water.

Perla Benghouzi [1], Lila Louadj [1,2], Maylis Garnier [1,2], Aurélia Pagani [1,2], Jérôme Fresnais [1], Carlo Gonzato [3], Michèle Sabbah [2] and Nébéwia Griffete [1,*]

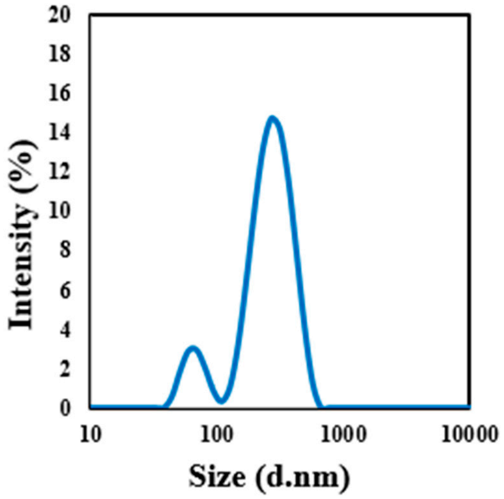

Figure S1. Dynamic light scattering (DLS) of the MIP after two months

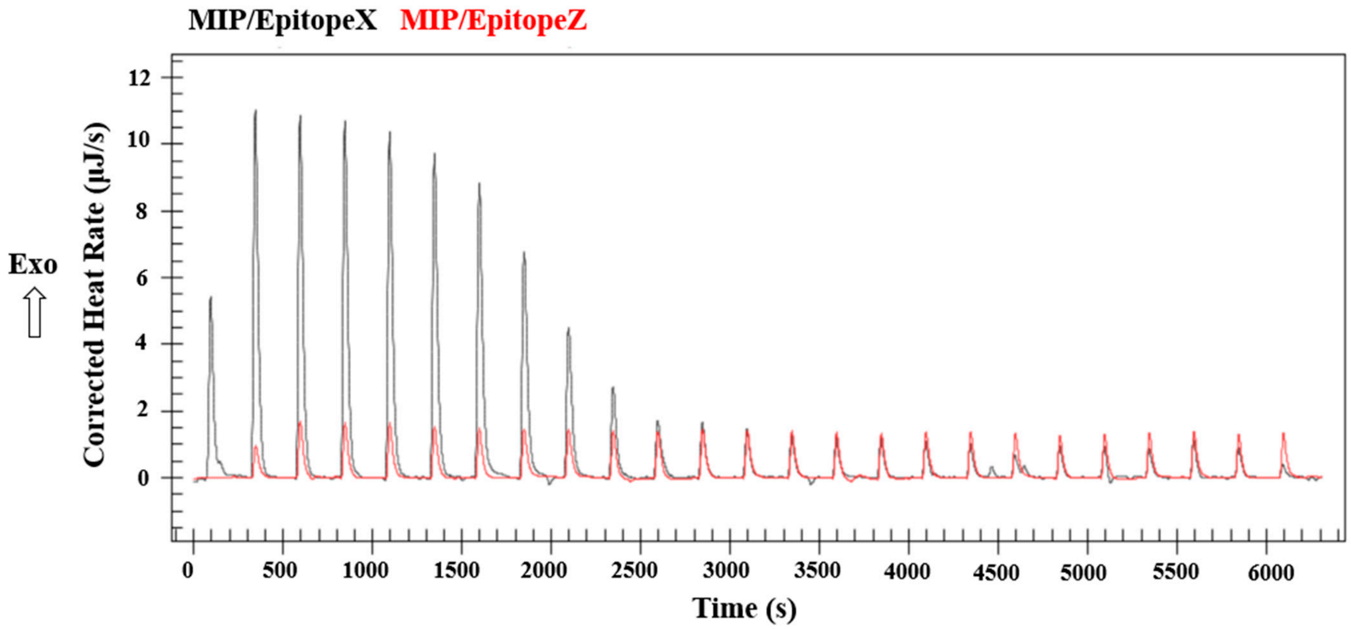

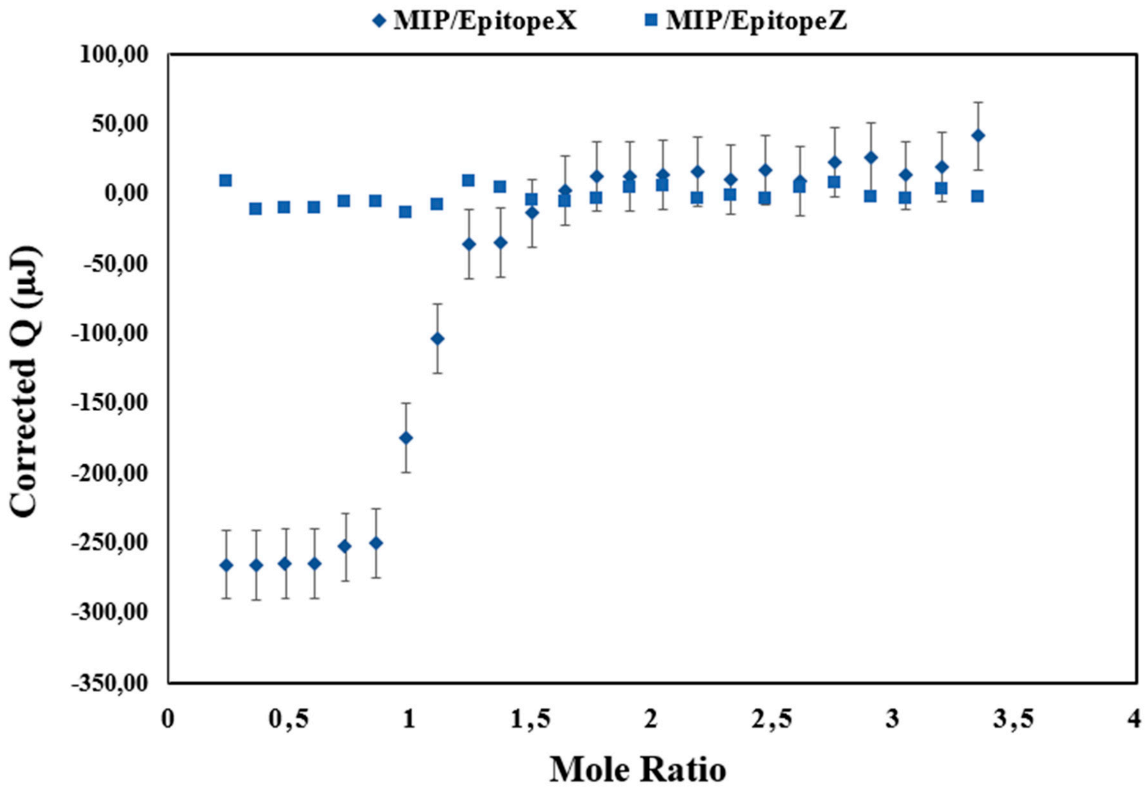

Figure S2. Calorimetric data for binding kinetics between MIP/epitope X and the MIP with another epitope Z from the same protein. Top: raw data from exothermic binding after continuous titration of MIP in epitope solution. Bottom: results calculated from the raw data as a function of the molar ratio of MIP to epitope (the first value was excluded from analyze).
